# Green Synthesis of Red Fluorescent Graphene Quantum Dots Using *Withania somnifera* Leaves: Exploring Antidiabetic and Antioxidant Potential

**DOI:** 10.1155/ijbm/5841012

**Published:** 2025-02-18

**Authors:** Sudhir Kumar Kataria, Pooja Kadyan, Jaya Saini, Mohit Saharan, Ponnusamy Thillai Arasu

**Affiliations:** ^1^Department of Zoology, Maharshi Dayanand University, Rohtak, Haryana, India; ^2^Department of Chemistry, Wollega University, P.O. Box 395, Nekemte, Oromia, Ethiopia

**Keywords:** antidiabetic, antioxidant, graphene quantum dots (GQDs), green synthesis, phytochemical characterization, *Withania somnifera* (Ashwagandha)

## Abstract

In recent years, green synthesis methods for producing nanomaterials have gained significant interest due to their environmentally friendly nature and wide-ranging applications. The present study addresses a novel green synthesis of graphene quantum dots (GQDs) using leaves of *Withania somnifera.* The size, morphology, and stability of the green-synthesized GQDs were characterized using TEM, UV-Visible spectroscopy, Fluorescence spectrophotometer, XRD, and DLS. The bio-functional properties of the GQDs were investigated, with a focus on their antidiabetic and antioxidant capabilities. Their antidiabetic activity was assessed by examining their ability to inhibit α-amylase and α-glucosidase enzymes, which play a crucial role in glucose metabolism. Additionally, their antioxidant properties were evaluated using DPPH● scavenging assays, highlighting their effectiveness in neutralizing free radicals. The findings revealed that the synthesized GQDs outperformed the original leaf extract in both antioxidant activity and enzyme inhibition. The study revealed that the leaf extract exhibited higher IC_50_ values for inhibiting DPPH (78.508 ± 5.71), α-amylase (161.909 ± 6.188), and α-glucosidase (133.345 ± 7.328) compared to synthesized GQDs, which showed lower IC_50_ values of 72.74 ± 5.9, 137.966 ± 6.95, and 122.084 ± 5.478, respectively. The findings indicate that *Withania somnifera* derived GQDs hold significant potential for medical applications, warranting further investigation into their therapeutic efficacy. This study offers a comprehensive analysis of the fundamental biological properties of GQDs, addressing the dual challenges of antidiabetic and antioxidant activity.

## 1. Introduction

Every population on the planet, regardless of location—including rural areas of middle- and low-income nations—has diabetes. According to WHO projections, diabetes is expected to rank seventh among the leading causes of death by 2030 [1]. Diabetes Mellitus (DM) is a prolonged metabolic illness marked by elevated levels of blood glucose (hyperglycemia) and irregular metabolism of fat, protein, and carbohydrate [2–4]. Over 90% of all diabetic patients have type 2 (NIDDM, noninsulin-dependent), among the most prevalent endocrine illnesses of the twenty-first century. NIDDM is marked by the body's incapability to respond to insulin [5]. Diabetes can be lessened in severity by preventing T2DM and treating all diabetes forms with appropriate treatment and an early diagnosis. One major contributing factor to the development of T2DM is postprandial hyperglycemia [4–6]. A treatment approach for decreasing postprandial hyperglycemia involves the ability of a drug or food plan to inhibit the hydrolysis of carbohydrates by enzymes such as α-amylase and α-glucosidase, delaying the production or assimilation of glucose [7]. One of the main ingredients of pancreatic fluid is α-amylase, which is produced by acinar cells of pancreas and released into the duodenum. α-amylase breaks down the starch in the duodenum to produce maltose, which brush-border membrane enzymes like beta-glucosidase then hydrolyze to produce absorbable monosaccharides [8]. There is a class of enzymes known as B-glucosidases found on the brush edge of enterocytes that line the intestinal villi [9]. The β-1,4 glycosidic link of several glycoconjugates, such as glucosides and oligosaccharides, can be hydrolyzed by it to generate glucose, which is the terminal, nonreducing β-D-glucosyl residues [10].

Furthermore, oxidation reactions are necessary for cell survival [5, 11, 12]. A free radical consists of an unpaired (free) electron with spin, a property of quantum mechanics. Due to its open shell nature, such an entity usually exhibits great reactivity [13]. Free radicals are usually considered to be linked to oxidative stress [13, 14]. When a cell's mitochondria create too many reactive oxygen species (ROS), it can lead to oxidative stress. Free radicals accumulate in biological systems and are known to cause a variety of degenerative diseases, including acute inflammation, carcinogenesis, hypertension, diabetes, acute renal failure, preeclampsia, atherosclerosis, mutagenesis, aging, and cardiovascular disorders [15].

Researchers are trying hard to identify new and alternative antioxidant molecules with less side effects because of the hazardous consequences of these synthetic additives [16]. Carbon-based nanostructures with unique characteristics may provide novel approaches [17, 18].

Recent years have seen a rise in the use of nanotechnology, which has had a significant influence on several scientific fields [19]. Their unique quantum properties and nanomaterials have provoked a lot of interest. The synthesis of nanoscale metals using different chemical, biological, and physical methods is the subject of substantial current study [17, 20–22]. Environmentally friendly synthesis techniques are gradually replacing both chemical and physical methods due to the adoption of advanced technology and analysis conditions, despite challenges involving excessive energy consumption, costly and tedious procedures, risks that demand extreme pressures and temperatures, and the release of potentially harmful substances [17, 23]. Contrarily, using chemicals is avoided in the green synthesis method. They are beneficial to the environment and are sturdy, one-step, and easy to use. Plant extracts and microorganisms (fungi, bacteria, and algae) work as agents in green synthesis, which facilitates the production of GQDs, due to their reducing and antioxidant properties [24].

Because of their unique qualities, carbon-based nanomaterials provide novel approaches to diabetic treatment [25]. Numerous new study fields have emerged as a result of recent developments in nanotechnology and nanoscience [17, 24]. Traditional carbon-based nanomaterials have become more well-known among them, including graphite, fullerenes, diamonds, carbon quantum dots, carbon nanotubes (CNTs), graphene oxide (GO), carbon dots, and GQDs. These materials are valuable because of their electrical, optical, and catalytic qualities, which are applied in the energy, environmental, and medical fields [24, 26]. Researchers in a variety of sectors have been enthralled by carbon-based materials because of its remarkable qualities, which include vast surface areas, minimal toxicity, superb conductivity, long-term stability, and customizable designs [17, 24]. Researchers' interest in the rapidly emerging field of graphene quantum dots (GQDs) has grown significantly. This is because of their photocatalytic activity, high hydrophobicity, photostability, cost-effectiveness, low cytotoxicity, chemical accessibility, steady fluorescence, and photoluminescence qualities [24–26]. *Withania somnifera,* often known as “Indian winter cherry,” “Indian ginseng,” or “Ashwagandha,” is a plant that has been extensively utilized for millennia in many parts of South Asia. It is a species of the family Solanaceae [27]. It is a well-known herbal Rasayana in Ayurveda and is called “Sattvic Kapha Rasayana” [27]. This study describes the environmentally friendly synthesis of GQDs with the use of microwaves and *Withania* leaves as stabilizing and reducing agents. There are several benefits of using plant leaves to produce GQDs, such as their widespread availability, affordability, safety, removal of hazardous chemicals, and the existence of several metabolites that support GQD synthesis.

## 2. Materials and Methods

### 2.1. Plant Material Collection and Identification

The leaves of *Withania somnifera* were picked in the month of February 2024 from the adjacent areas of Bhagwad Bhakti Hermitage, Rampura, Sector 15, Rewari, Haryana. The plant was identified using the *Flora of Haryana*. After giving the leaves, a thorough washing to get rid of any dust, they were permitted to dry in the shade for a period of 12 days. Once the leaves were dried, they were powdered into a coarse powder.

### 2.2. Chemicals and Reagents

α-glucosidase from *Saccharomyces cerevisiae* (EC number-232-604-7), and α-amylase from porcine pancreas (EC number 232-565-6)) were ordered from Sigma-Aldrich. di-nitrosalicylic acid (DNS; EC number 210-204-3), *p*-nitrophenyl-α-D-glucopyranoside (pNPG; EC number-219-661-3), di-methyl sulphoxide, sodium potassium tartrate tetrahydrate, sodium hydroxide, ascorbic acid, sodium carbonate (Mw. 105.98 g moL^−1^), sodium phosphate, 2,2-diphenyl—picrylhydrazyl (DPPH), ethanol (99.9%), methanol, sulfuric acid (98%), and starch were purchased from Hi-Media, Mumbai, distilled water, Milli-Q water.

### 2.3. Apparatus and Instruments

Hot plate with magnetic stirrer, mortar and pestle, graduated cylinders, beakers, micropipette, thermometer, electric grinder, domestic microwave (900W), centrifuge machine, UV-Visible spectroscopy (Shimadzu, UV 3600plus), XRD (Rigaku, Smart lab 3 KW), FESEM-EDX (JEOL, 7610F plus), HRTEM (TECNAI, 200kv), FTIR (Bruker), Zeta potential (Malvern, Nano ZS), Fluorescence Spectrophotometer (HORIBA).

### 2.4. Synthesis Protocol of Plant Leaf Extract and GQDs

The maceration process was employed for extraction of dried leaf powder [28]. For this, 20 g of leaf powder were dissolved in 200 mL of ethanol and let to stand for 3 days at room temperature. The leftover solution was then filtered and transferred to the petri dish and oven-dried at 40°C. The percentage yield of the extract was calculated using equation [1]:

(1) $$\begin{array}{c} \text{Yield }\left(\%\right)=\left(\frac{W1}{W2}\right)\times 100, \end{array}$$

where *W*1 represents the weight of the crude solid extract, and *W*2 is the weight of the powdered plant material used. The total yield of the extract was determined to be1.080 g, corresponding to 5.40%. For future use, the leaf extract was scraped off the petri dish and refrigerated at 4°C in Eppendorf tubes.

**Figure 1:**
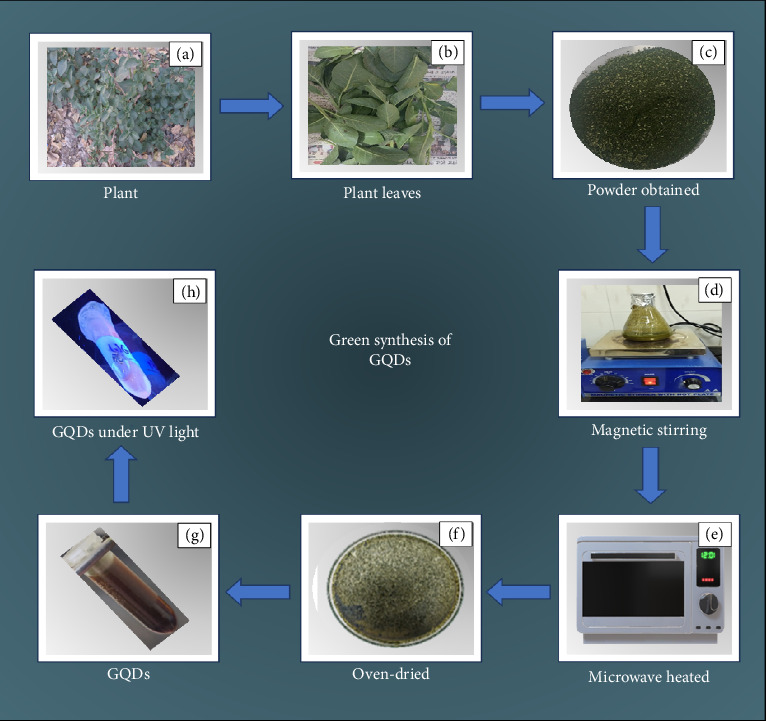
Green synthesis of GQDs.

The green synthesis of GQDs was conducted following the methodology described by Kumawat et al., with minor adjustments [22, 24, 29]. 25 gm of *Withania somnifera* leaf powder were dissolved in 250 mL of absolute alcohol and continually agitate the ingredients at room temperature for 4 h by employing a magnetic stirrer. To get a clear solvent, the extract was centrifuged at 5000 rpm for 12 min. A rotary evaporator was used to concentrate the solvent, and the resulting thick slurry was mixed with Milli-Q water. This mixture was then heated in a domestic microwave (900 W) for 6 min (Figure 1). The residue was dispersed in 99.9% ethanol and filtered through a 0.2 μm syringe filter to obtain pure GQDs which yield total GQDs of 2.716 g (i.e., 13.58%).

### 2.5. DPPH Free Radical Scavenging Assay

Free radicals are produced by a variety of biological processes. The elimination of free radicals that arise from the interaction of biomolecules with molecular oxygen is a leading area of nanomaterials research and application. The test for DPPH free radical scavenging was employed to assess the antioxidant efficacy of GQDs and leaf extract [14, 24]. 0.2 mL of a 1 mM DPPH solution in methanol was mixed with 2 mL of test samples (20–100 μg/mL). After 30 min of incubation in the dark, the absorbance was measured at 517 nm. Using equation (2), the capacity to scavenge the DPPH radical was determined.

(2) $$\begin{array}{c} {\text{DPPH}}^{●}\text{ scavenging }\left(\%\right)=\frac{\left(\text{Absorbance of control }–\text{ Absorbance of sample}\right)}{\text{Absorbance of control}}\times 100. \end{array}$$

An inhibition curve was plotted to determine the IC_50_ value, which was expressed as the mean ± standard deviation (SD) based on the results of three independent experiments.

### 2.6. In Vitro Antidiabetic Assay

#### 2.6.1. Alpha-Amylase Inhibitory Assay

Alpha-amylase inhibition activity of leaf extracts was conducted using the norm method with minor modifications [30]. After dissolving 7 mg of leaf extract in 7 mL of 10% DMSO (conc. 1 mg/mL), the extract was then diluted in 20 mM phosphate buffer (6.9 pH), resulting in a concentration range from 12.5 to 400 μg/mL. Then, 1 mL of extract (with varying conc.) was mixed with 1 mL of alpha amylase solution (1g alpha-amylase enzyme in 100 mL phosphate buffer, 20 mM; pH 6.9) and incubated at 25°C for 30 min. Thereafter, add 1 mL of 1% starch solution and incubated for 10 min at 37°C. At last DNS solution (24 g of NaKC_4_H_4_O_6_.4 H_2_O was dissolved in 16 mL of 2M NaOH and 40 mL of 96 mM DNS) was added and boiled for 10 min at 85–90°C to hault the reaction. The reaction mixture was mixed well and the absorbance was measured at 540 nm. For control, the plant extract was replaced with 1 mL of DMSO solution; and for blank, DNS was added prior to the starch solution. All other procedures were done exactly the same as for the test.

#### 2.6.2. Alpha-Glucosidase Inhibitory Assay

Alpha-glucosidase inhibition activity of leaf extracts was conducted using the standard procedure with minor modifications [30]. After dissolving 7 mg of leaf extract in 7 mL of 10% DMSO (conc. 1 mg/mL), the extract was then diluted in 50 mM phosphate buffer (6.8 pH), resulting in a concentration range of from 12.5 to 400 μg/mL. Then, 1 mL of extract (with different conc.) was mixed with 1 mL of alpha glucosidase solution (1 gm alpha-amylase enzyme in 100 mL phosphate buffer, 50 mM; pH 6.9) and incubated for 15 min at 37°C. Thereafter, add 1 mL of pNPG (5 mM) solution and incubated at 37°C for 20 min. Finally, the reaction was ceased by adding 2 mL of 0.1M Na_2_CO_3_. The enzyme's activity was quantified by measuring the amount of p-nitrophenol that was released from the pNPG substrate. The absorbance was measured at 405 nm. For control, the plant extract was substituted with 1 mL DMSO solution. All other procedures were done exactly the same as for the test.

Acarbose, a well-known antidiabetic drug, was employed as a positive control to compare the results. Every test was administered three times. Equation (3) was used to obtain the percentage inhibition.

(3) $$\begin{array}{c} \%\text{ Inhibition}=\frac{\left(\text{Absorbance of control }–\text{ Absorbance of sample}\right)}{\text{Absorbance of control}}\times 100. \end{array}$$

### 2.7. Statistical Analysis

The mean value ± SD obtained from three independent experiments, each carried out in triplicate, are displayed in the findings. The statistical analysis was performed with version 10.2.3 of GraphPad Prism 10. One-way ANOVA was used to evaluate significance at *p* < 0.05, and Tukey's multiple comparison tests with a 95% confidence interval were used as post hoc testing. ⁣^∗^*p* < 0.05, ⁣^∗∗^*p* < 0.01, and ⁣^∗∗∗^*p* < 0.001 were used to indicate the significance levels.

## 3. Results and Discussion

### 3.1. Optical Properties

**Figure 2:**
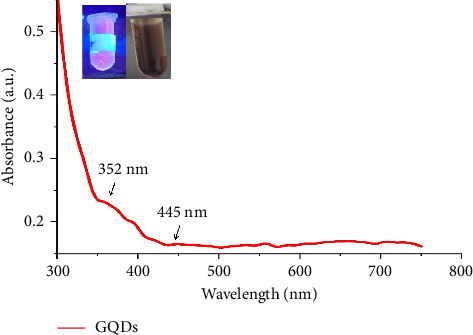
Absorption spectrum of GQDs.

In the UV-Visible spectrophotometric analysis, maximum absorption peaks were found at 352 nm which can be attributed to the *C*=O bonds *n* − *π*^∗^ transition whereas the peaks recorded at 445 nm might be corresponds to *n* − *π*^∗^ of sp^2^ domains (Figure 2). These results align with with previous studies describing GQDs [24, 31, 32].

A Tauc plot was used to examine the optical attributes of semiconducting materials to estimate their bandgap energy. In the visible spectrum to near-infrared (NI) spectrum, the nanoparticles displayed a plasmon resonance band due to the group oscillation of conduction electrons inside the particles. The Tauc and Menth method was used to compute the bandgap energy, as described by equations (4) and (5).

(4) $$\begin{array}{c} {\left(\propto h\nu \right)}^{2}=C\left(h\nu -E_{g}\right), \end{array}$$

(5) $$\begin{array}{c} \alpha =\frac{1}{t}\ln\left(\frac{T}{100}\right). \end{array}$$

**Figure 3:**
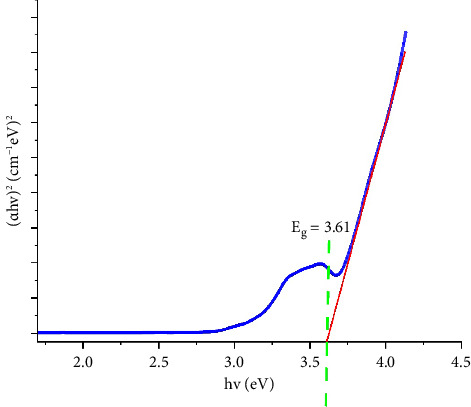
Tauc plot of GQDs.

The Beer–Lambert law was used to calculate the absorption coefficient (*α*) and plot it versus photon energy (*hv*). The resultant curve was then evaluated (Figure 3). In conclusion, the optical characteristics of carbon nanoparticles were shown by the Tauc plot. The nanoparticles' band gap energy (eV) was 3.61 eV. The optical and electrical characteristics of carbon nanoparticles can be designed and optimized for a variety of uses using the Tauc plot values. The findings of the tauc plot correspond to the red region's observed in luminescence spectra, suggesting that these GQDs have a broad bandgap. A material's bandgap energy corresponds with its optical and electrical characteristics, and semiconductors with strong luminescence are frequently found to have a broad bandgap energy.

### 3.2. Photoluminescence Analysis

At 650–750 nm, in the red spectrum region, the GQDs displayed a prominent luminescence band. A strong peak is produced at 350 nm due to acceleration of electrons from the valence band to the conduction band. The modest variation in band gap and intensity between chemical production and sustainable production could be explained by the biomolecules present on the surface of GQDs. This suggests that the produced GQDs have superior optical qualities, which qualify them for a number of uses. Figure 2 illustrates the colour of the nanoparticle solution as brown when exposed to sunlight light and a bright red fluorescent when exposed to ultraviolet light. Additionally, a notable peak of absorption is seen in the sample in the NI region (NIR) at around 660 nm. This peak corresponds to the red, intense fluorescence that the samples display when exposed to UV light. The red fluorescence highlights the immense potential of these generated CNPs in therapeutic applications, as the higher wavelength tends to locate deeply seated tissues at the targeted region.

### 3.3. Microscopic Study

**Figure 4:**
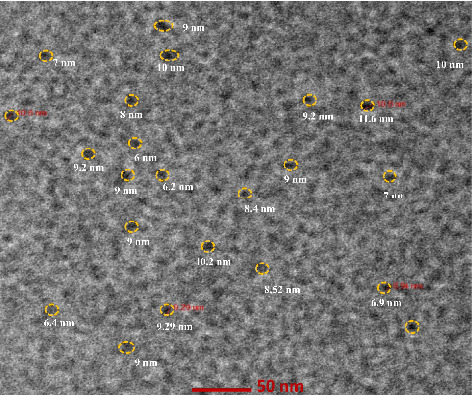
TEM image of GQDs at 50 nm scale.

**Figure 5:**
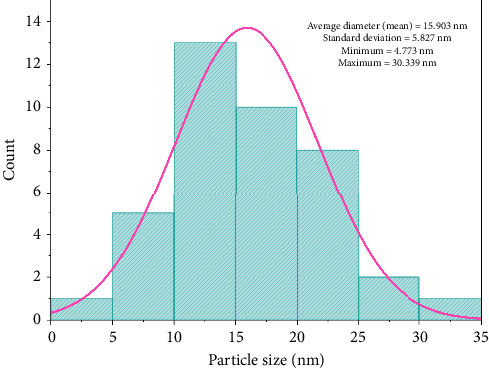
Histogram image of GQDs.

TEM study validated the structural morphology of GQDs. To prepare the sample, GQDs were dissolved in ethanol, and sonication was used to lessen aggregation. A little drop of the mixture was placed on a copper grid. After drying, the samples were subjected to an analysis at a resolution of 0.24 nm and 200 kV acceleration voltage.  Figure 4 illustrates the spherical appearance of the GQDs. The histogram and particle size distribution curve (Figure 5) plotted from TEM images revealed the average size of GQDs to be 15.903 ± 5.827 nm.

### 3.4. Zeta Potential

**Figure 6:**
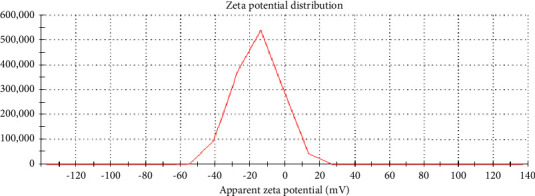
Zeta potential of GQDs.

The Zeta potential measurement of the GQDs revealed value of −15.7 mV (Figure 6). A high Zeta potential (negative or positive) signifies strong repulsion between particles, thus preventing aggregation. Because of the many hydroxyl and carboxyl groups that are present on their surface and contribute to their stability and dispersibility in aqueous solutions, the Zeta potential study showed that the GQDs were negatively charged [24].

### 3.5. X-Ray diffraction (XRD) Analysis

The materials have been investigated using powdered XRD within the 2*θ* range of 10°–80°. By using an anode of Cu-Kα radiation with a wavelength of 1.5406 Å was employed at scanning rate of 2°/minute, to elucidate the crystalline structure of the synthesized GQDs at the atomic level. The interplanar spacing, or d-spacing, for the XRD analysis was calculated using Bragg's Law, which establishes a link between the wavelength of the incident X-rays, the diffraction angle, and the crystal's d-spacing, as described in equations (6) and (7). Furthermore, the Debye–Scherrer equation was applied to estimate the particle size of the synthesized GQDs, as detailed in the following equation.

(6) $$\begin{array}{c} n\lambda =2\text{dsi}n\theta , \end{array}$$

where *n* represents the order of reflection (set to 1 for this analysis), *λ* denotes the X-ray wavelength (1.5406 Å), and *θ* is the Bragg angle.

(7) $$\begin{array}{c} D=\frac{0.9\lambda }{\beta \;\cos\;\theta }, \end{array}$$

where *D* is the crystallite size of the nanoparticles, *K* is the Scherrer constant, typically ranging from 0.9 to 1, *θ* is Bragg's angle in radians, *λ* is the wavelength of the X-ray, and *β* is the full width at half maximum.

The XRD pattern's distinctive peaks illustrate the presence of secondary metabolites, which affect the GQDs' stability and purity [22, 33]. It was necessary to utilize further plant extracts and to check stirring times because these nanoparticles show crystallinity and have a quantifiable size. The crystal structure and crystallite size of nanoparticles can be ascertained by looking at the location and intensity of the peaks in the XRD pattern. It also suggests the size range of the crystallites within nanomaterial. The breadth of the observed peaks is inversely correlated with the size of the crystallites; wider peaks denote smaller crystallites. The crystallite size range in this study was determined to be between 16.88 nm, which the histogram also validated. Understanding the material's physical and chemical properties requires knowledge of this information. A nanomaterial's mechanical strength, electrical characteristics, or catalytic activity can all be improved by adjusting its size and crystal structure.

**Figure 7:**
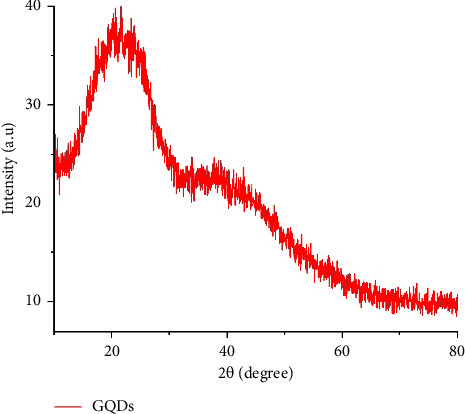
XRD of GQDs.

The XRD pattern of the synthesized GQDs revealed a prominent peak at 22.3° (Figure 7), confirming the presence of GQDs. This discovery, which is consistent with other observations that found a distinctive peak at 25.80°, supports the theory that the current synthesized GQDs have a graphitic nature [24, 31, 32, 34].

### 3.6. DPPH Radical Scavenging Assay

**Figure 8:**
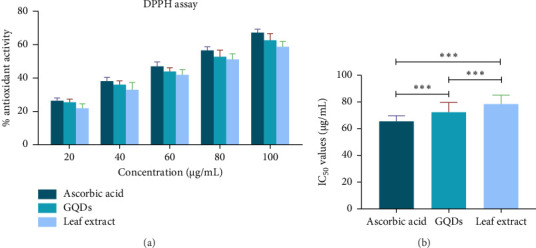
(a) Antioxidant potential of *Withania* synthesized GQDs, *Withania* leaf extract, and standard. (b) IC_50_ value of *Withania* GQDs, *Withania* leaf extract, and standard.

The DPPH radical was scavenged by antioxidants by proton donation, which led to the DPPH being dropped. The primary signal, the colour shift, shows how much free radical scavenging has occurred. The synthesized GQDs DPPH free-radical scavenging activity (antioxidant properties) was lower than ascorbic acid, but it was higher than leaf ethanolic extract (Figures 8(a) and 8(b)). Previous research suggested that the samples exhibited significant DPPH free radical scavenging activity [24]. The movement of hydrogen from GQD surfaces to DPPH is one potential radical scavenging pathway. The presence of carboxyl's (-COOH), hydroxyl's (-OH), and amino groups (-NH2 and -NH) promotes hydrogen atoms transfer and the DPPH reduction. IC_50_ refers to the amount of an antioxidant-containing material needed to scavenge half of the initial radicals.

The IC_50_ value are inversely correlated with the antioxidant and free radical scavenging activity. A compound's potential to scavenge DPPH is suggested by a lower IC_50_ value, which also indicates more antioxidant activity. GQDs outperform the crude extract in terms of antioxidant activity because of their lower IC_50_ value.

### 3.7. In Vitro Antidiabetic Assay

#### 3.7.1. Alpha-Amylase Inhibitory Activity

**Figure 9:**
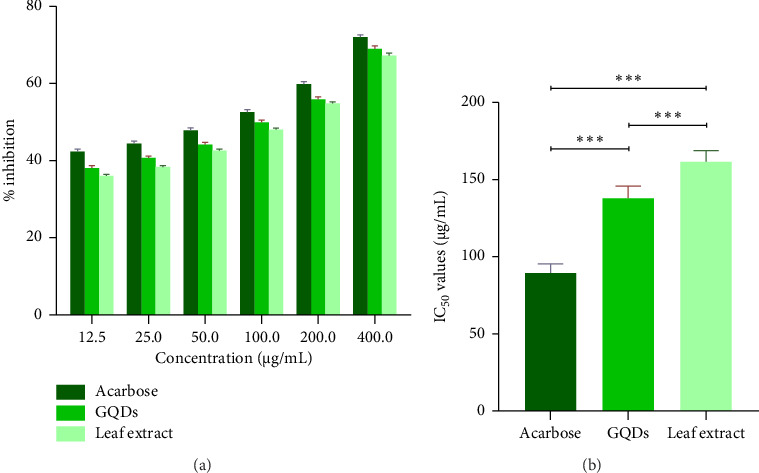
(a) Alpha-amylase inhibitory efficacy of *Withania* GQDs and *Withania* leaf extract. (b) IC_50_ value of *Withania* leaf extract, GQDs and standard acarbose. Results as mean ± standard error; ⁣^∗∗∗^(*p* ≤ 0.001), ⁣^∗∗^(*p* ≤ 0.01), ⁣^∗^(*p* ≤ 0.05).

The α-amylase inhibitory activity was measured using a colorimetric method with the DNS reagent. α-amylase acts on starch and converts into maltose. Alkaline DNS (pale yellow) turns maltose into orange-red colour. The strength of the colour corresponds to the quantity of maltose. Leaf extracts and GQDs show alpha-amylase inhibition, which inhibits starch from breaking down into maltose and lowers absorbance. From the experiment, it was revealed that synthesized GQDs have greater ability to suppress alpha-amylase activity than ethanolic leaf extract of *Withania somnifera* (Figures 9(a) and 9(b)). GQDs outperform the crude extract in terms of antidiabetict activity because of their lower IC_50_ value.

#### 3.7.2. Alpha-Glucosidase Inhibitory Activity

The α-glucosidase inhibitory activity was assessed using a colorimetric method employing the pNPG reagent. When α-glucosidase encounters pNPG, it transforms it into pNP, which has a light-yellow colour. The intensity of the color corresponds to the amount of pNP in the sample.

**Figure 10:**
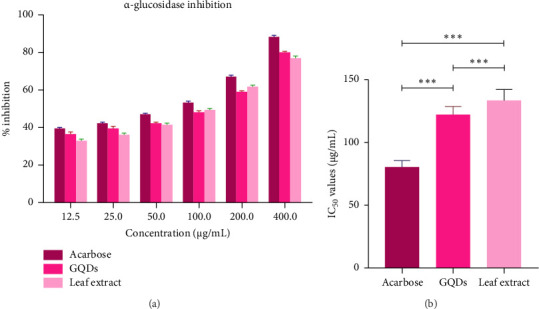
(a) Alpha-glucosidase inhibitory potential. (b) IC_50_ value of *Withania* leaf extract, GQDs, and acarbose. Results as mean ± standard error; ⁣^∗∗∗^(*p* ≤ 0.001), ⁣^∗∗^(*p* ≤ 0.01), ⁣^∗^(*p* ≤ 0.05).

Alpha-glucosidase is inhibited by the leaf extracts and GQDs, which stops pNPG from breaking down into pNP and lowers absorbance. From the experiment, it was revealed that synthesized GQDs have greater ability to suppress alpha-glucosidase activity than ethanolic leaf extract of *Withania somnifera* (Figures 10(a) and 10(b)). GQDs outperform the crude extract in terms of antidiabetic activity because of their lower IC_50_ value.

## 4. Conclusion

This work illustrates the effective phytosynthesis of GQDs using plant extracts, providing a safe and environmentally beneficial substitute for traditional nanomaterial production techniques, which generally include dangerous chemicals. HRTEM, UV-Visible, XRD, DLS were used for the characterisation of phyto-mediated GQDs to verify their size, shape, and morphology. The particle sizes of the GQDs were less than 20 nm, according to the size distribution analysis performed on the TEM data. Furthermore, it was shown that the phyto-mediated GQDs' antioxidant and antidiabetic efficacy surpassed that of the crude plant extract, suggesting increased potential for biomedical applications. These findings highlight the adaptability and effectiveness of GQDs and open the door to further research into their usage in a variety of applications, including medication delivery and bioimaging. From a nutraceutical and pharmaceutical development perspective, these results provide important new insights into the potential of traditional therapeutic herbs. The potential of employing phytochemicals in the synthesis of advanced materials is highlighted by the proven effectiveness of plant-based GQDs, underscoring their function in the development of long-lasting and potent medicinal medicines. This study creates new opportunities for the investigation and application of conventional botanical resources in the creation of novel products that improve health.

## Data Availability

The data supporting this study will be available on request.
